# Retraction of: Tiny Bryophytes: Nature’s Hidden Reservoirs of Powerful Anti-Cancer Compounds

**DOI:** 10.1093/plcell/koaf280

**Published:** 2026-01-02

This is a retraction of: Ruturaj S Shete, Maruti J Dhanavade, Mudasir A Dar, Shashikant J Chavan, Tiny Bryophytes: Nature’s Hidden Reservoirs of Powerful Anti-Cancer Compounds, *The Plant Cell*, 2025; https://doi.org/10.1093/plcell/koaf268

An unrefereed manuscript was erroneously transmitted to publication by ASPB journal production staff, so it is being retracted by the journal Editor-in-Chief. This retraction is due solely to the error of the journal and in no way suggests any misconduct on the part of the authors. The journal and OUP apologize for any problems that the publication of this paper may have caused.

